# Reply to Swaika et al.

**DOI:** 10.1093/icvts/ivag159

**Published:** 2026-06-02

**Authors:** Makoto Okawara, Yukiko Nemoto, Yoshihisa Fujino, Fumihiro Tanaka

**Affiliations:** Department of Preventive Medicine and Community Health, School of Medicine, University of Occupational and Environmental Health, Japan, Kitakyushu, Fukuoka, 807-8555, Japan; Second Department of Surgery, University of Occupational and Environmental Health, Japan, Kitakyushu, Fukuoka, 807-8555, Japan; Department of Environmental Epidemiology, Institute of Industrial Ecological Sciences, University of Occupational and Environmental Health, Japan, Kitakyushu, Fukuoka, 807-8555, Japan; Second Department of Surgery, University of Occupational and Environmental Health, Japan, Kitakyushu, Fukuoka, 807-8555, Japan

We thank Swaika and colleagues for their insightful comments on our study comparing robotic-assisted thoracoscopic surgery (RATS) and video-assisted thoracoscopic surgery (VATS) for lung cancer using Japan’s Diagnosis Procedure Combination (DPC) database.^1^^,^^2^ Many of the points raised by the correspondents align with the limitations and interpretative caveats discussed in our original paper, including outcome definition, residual confounding, between-centre differences and missing data.

First, we agree that administrative codes cannot fully distinguish all clinical situations leading to postoperative ventilatory support, including planned postoperative management, transient ventilatory support and clinically significant respiratory failure. However, our end-point was not routine intraoperative ventilation. It was defined as the re-initiation of non-invasive or invasive ventilation after extubation between postoperative days 1 and 30.^2^

Second, institutional practice may influence postoperative mechanical ventilation. As stated in the original paper, we therefore used multilevel models incorporating hospital-level random effects and conducted sensitivity analyses, including an analysis restricted to hospitals that performed both VATS and RATS during the study period. These methods cannot completely eliminate residual facility-level differences, particularly when postoperative management protocols vary between hospitals, but they were intended to reduce confounding due to facility-specific clinical practice within the available data.

Third, unmeasured confounding is an important limitation of observational studies using administrative data. The DPC database does not include detailed clinical variables such as pulmonary function, tumour size, conversion to open surgery, surgeon experience, or detailed perioperative ventilation strategies. For this reason, our interpretation was cautious: we described the findings as associations rather than definitive causal effects and reported both relative and absolute effect measures to avoid overemphasizing a statistically significant but clinically small difference.

Randomization is the most robust design-based method for balancing measured and unmeasured baseline confounders. However, surgical randomized trials also require careful consideration of generalizability, as they are often conducted in selected centres, by experienced surgeons, under standardized perioperative protocols and in carefully selected patients.^3^^,^^4^ Therefore, randomized trials and nationwide administrative database studies should be viewed as complementary rather than competing sources of evidence. While randomized trials are indispensable for estimating efficacy under controlled conditions, real-world database studies can describe outcomes across heterogeneous patients, surgeons, hospitals, and perioperative care settings.^5^

We also appreciate the comments regarding analgesic methods and adjustment for anaesthesia duration. These variables may partly lie on the causal pathway between surgical approach and postoperative respiratory outcomes. This additional adjustment was exploratory and was not intended to redefine the primary estimand or to estimate the total causal effect. Similarly, complete-case analysis relies on assumptions regarding missing data. Although multiple imputation yielded largely consistent results, we cannot completely rule out missingness related to patient complexity or facility-specific recording practices.

Overall, the points raised by Swaika et al reinforce the cautious interpretation presented in our original paper. Our study should be interpreted as a nationwide real-world evaluation rather than definitive causal evidence. The findings suggest comparable overall perioperative safety between RATS and VATS, while a small absolute increase in postoperative mechanical ventilation warrants further evaluation in prospective studies.
